# Comparison of Bioactive Compounds and Antioxidant Activities of *Maclura tricuspidata* Fruit Extracts at Different Maturity Stages

**DOI:** 10.3390/molecules24030567

**Published:** 2019-02-04

**Authors:** Dae-Woon Kim, Won-Jae Lee, Yoseph Asmelash Gebru, Han-Seok Choi, Soo-Hwan Yeo, Young-Jae Jeong, Seung Kim, Young-Hoi Kim, Myung-Kon Kim

**Affiliations:** 1 Department of Food Science and Technology, Chonbuk National University, Jeonju 54896, Jeonbuk, Korea; eodns3344@gmail.com (D.-W.K.); iwjae@foodpolis.kr (W.-J.L.); yagebru@gmail.com (Y.A.G.); yhoi1307@hanmail.net (Y.-H.K.); 2 Fermented Food Science Division, National Institute of Agricultural Sciences, Rural Development Administration (RDA), Wanju 55365, Jeonbuk, Korea; coldstone@korea.kr (H.-S.C.); yeobio@korea.kr (S.-H.Y.); 3 Agency for Korea National Food Cluster (AnFC), Iksan 54576, Jeonbuk, Korea; gentlejeong@foodpolis.kr; 4 Department of Food Science and Biotechnology, Gwangju University, Gwangju 61743, Korea; seungk@gwangju.ac.kr

**Keywords:** *Maclura tricuspidata* fruit, HPLC-QTOF-MS, HPLC-DAD, polyphenolic compounds, parishin derivatives, maturity stages, antioxidant activity

## Abstract

*Maclura tricuspidata* fruit contains various bioactive compounds and has traditionally been used in folk medicine and as valuable food material in Korea. The composition and contents of bioactive compounds in the fruit can be influenced by its maturity stages. In this study, total phenol, total flavonoid, individual polyphenolic compounds, total carotenoids and antioxidant activities at four maturity stages of the fruit were determined. Polyphenolic compounds were analyzed using high-pressure liquid chromatography-quadrupole time-of-flight mass spectrometry (HPLC-QTOF-MS) and HPLC. Among 18 polyphenolic compounds identified in this study, five parishin derivatives (gastrodin, parishin A, B, C, E) were positively identified for the first time in this plant. These compounds were also validated and quantified using authentic standards. Parishin A was the most abundant component, followed by chlorogenic acid, gastrodin, eriodictyol glucoside, parishin C, parishin E and parishin B. The contents of all the polyphenolic compounds were higher at the immature and premature stages than at fully mature and overmature stages, while total carotenoid was found to be higher in the mature and overmature stages. Overall antioxidant activities by three different assays (DPPH, ABTS, FRAP) decreased as maturation progressed. Antioxidant properties of the fruit extract are suggested to be attributed to the polyphenols.

## 1. Introduction

*Maclura tricuspidata* (Carr.) Bur (formerly known as *Cudrania tricuspidata*), which belongs to the Moraceae family, is a thorny tree native to East Asia, including China, Japan, and Korea. The leaves, root, stem, and fruit of this plant have been used in traditional Korean herbal medicines to treat jaundice, hepatitis, neuritis, and inflammation [1]. Over the last two decades, several beneficial effects of *M. tricuspidata* extracts have been reported; they include anticancer [2,3], anti-inflammatory [4], antioxidant [5,6], oxidative stress-induced neurotoxicity [7], anti-obesity and anti-diabetes properties [8]. Moreover, a variety of bioactive compounds such as prenylated xanthones, phenolic acids, and flavonoids have already been identified from its leaves, root, stem, and fruit [9,10,11]. These compounds were also reported to have antitumor [11], antibacterial [12], antioxidant [13,14], neuroprotective [1], cytotoxic [13,15], anti-inflammatory [16], hepatoprotective [17], gastroprotective [16], and α-glucosidase inhibition activities [18].

Polyphenols in a plant diet gained much interest mainly due to their antioxidant properties, which provide health benefits such as anti-inflammatory, anti-carcinogenic, anti-atherogenic, antithrombotic, immune-enhancement, and vasodilatory effects [19]. These compounds act as scavengers of reactive oxygen species such as hydroxyl, hydroperoxyl, and superoxide anion radicals, which are inevitably produced by the metabolic reactions of living systems [20,21]. These radical species attack lipids, proteins and DNA, damaging biological structures, including cell membranes, enzymes, and genetic material. This damage can cause various chronic diseases such as cancer, cardiovascular diseases, atherosclerosis, hypertension, diabetes, neurodegenerative disorders, rheumatoid arthritis, and aging [22].

Both the premature and fully mature fruits of *M. tricuspidata* have traditionally been used in Korea to make fresh juice, jam, wine, vinegar, and fermented alcoholic beverages. The cultivation area of this plant for fruit production has recently increased substantively and is to some extent being popularized. While interest in its extracts has steadily increased due to its diverse biological activities, little is known about the bioactive compounds of its fruit when compared to those of its leaves, root, and bark. Due to this, there is limited information available about variations in the level of total polyphenols, individual polyphenols, and antioxidant activities of the fruit at different maturity stages.

This study aimed at exploring the variations of total polyphenols, individual polyphenolic compounds, and antioxidant activities at different maturity stages of the *M. tricuspidata* fruit. We developed systematic methods of HPLC/Q-TOF-MS and reverse phase high performance liquid chromatography (RP-HPLC) with photo-diode array detection (DAD) to identify and quantify newly discovered compounds. We were able to identify five parishin derivatives (gastrodin, parishin A, B, C, E) for the first time.

## 2. Results and Discussion

### 2.1. Identification of Polyphenolic Compounds

Biological therapeutic compounds that are commonly plant secondary metabolites can be analyzed with various methods. A highly sensitive QTOF-MS method is being employed to identify trace constituents that are not detectable using the classical analytical methods. In this study, *M. tricuspidata* fruit was extracted with 70% aqueous methanol and the polyphenolic components of the extract were analyzed by HPLC/Q-TOF-MS in negative electrospray ionization (ESI) mode, whereby the compounds were characterized by the interpretation of their mass spectra obtained by the Q-TOF-MS analysis, also taking the mass spectral data from databases and the literatures into account.

**Figure 1:**
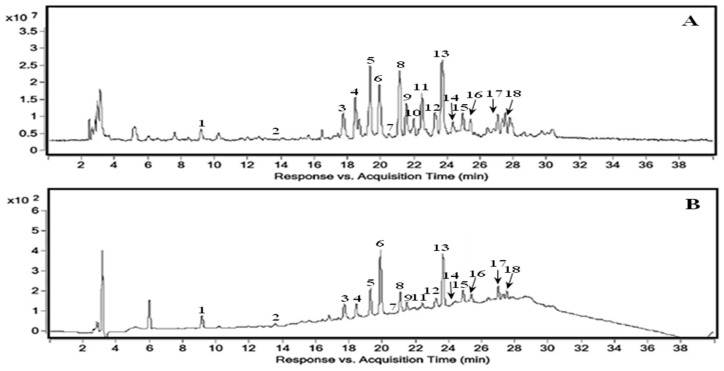
HPLC-QTOF-MS analysis of 70% methanol extract of *M. tricuspidata* fruit. (**A**) TIC; (**B**) DAD (220 nm).

The total ion current (TIC) chromatogram and HPLC-DAD-QTOF-MS chromatogram at 220 nm of 70% methanol extract isolated from fully mature *M. tricuspidata* fruit are shown in Figure 1A,B, respectively. The characteristics of either positively or tentatively identified compounds are listed in Table 1 along with their retention time, molecular mass, molecular formula, and error values (ppm) calculated by the software and identification results based on the database.

Among them, gastrodin (**1**) and four parishin analogues (**4, 9, 11, 13**) were positively identified for the first time in the fruit of this plant, whereas the other 10 compounds, including one flavanol (peak **3),** one flavanone **(6**), six flavonols (**7**, **10, 12, 16, 17, 18**), one anthocyanidin (**14**) and chlorogenic acid (**5**) have been previously reported in the plant [11].

In this respect, peak **3** (at 17.75 min) yielded [M − H]^−^ at *m*/*z* 465.1034 with MS fragment at *m*/*z* 303.0510 due to loss of a glucosyl moiety [M − H − glucose]^−^. This component was tentatively characterized as a taxifolin 3-*O*-glucoside, a component that has been previously reported in the twigs of *M. tricuspidata* [23]. Additionally, Peak **5** (at 19.35 min) showed [M − H]^−^ at *m*/*z* 353.0875. This compound was positively identified as chlorogenic acid according to its fragmentation pattern and appearance of a fragment at *m*/*z* 191.0562 [quinic acid − H] ^−^ due to the loss of caffeic acid, and by comparison of the HPLC retention time and UV profile with the authentic standard. This compound has also been previously reported in the leaf and fruit extracts of the plant [24]. Peak **6** (at 19.95 min) showed a molecular ion peak at *m*/*z* 449.1084 [M − H]^−^ with *m*/*z* 287.0558 due to loss of a glucosyl moiety [M − H − glucose]^−^. Accordingly, this compound was consequently identified as eriodictyol glucoside. The molecular ion peaks [M − H]^−^ of peak **7** (at 20.48 min) and **10** (at 22.02 min) were observed at *m*/*z* 625.1407 and *m*/*z* 609.1456, representing molecular ion peaks corresponding to rutin and quercetin 3-sophoroside, respectively [25]. Peak **12** (at 23.26 min) and **16** (at 25.43 min) were characterized as the isomers of the quercetin glucoside based on molecular ion peaks [M − H]^−^ at *m*/*z* 463.0875 and *m*/*z* 463.0875. Peak **14** was characterized as peonidin 3-glucoside based on [M − H]^−^ at *m*/*z* 461.1663. Peak **17** (at 27.06 min) and **18** (at 27.53 min) yielded molecular ion peaks [M − H]^−^ at *m*/*z* 287.0559 and *m*/*z* 447.0927, respectively. In line with this analysis and data from previous results [25,26], the molecular ions at *m*/*z* 287.0559 and *m*/*z* 447.0927 indicated that these compounds are dihydrokaempferol and kaempferol 3-*O*-glucoside. Both of these compounds have been previously identified in *M. tricuspidata* fruit extracts [26].

### 2.2. Identification of Parishin Derivatives

Among the 18 phenolic compounds identified, we selected parishin metabolite gastrodin (**1**) and four parishin analogues (**4, 9, 11, 13**) for further characterizations as they are identified in the plant for the first time.

**Figure 2:**
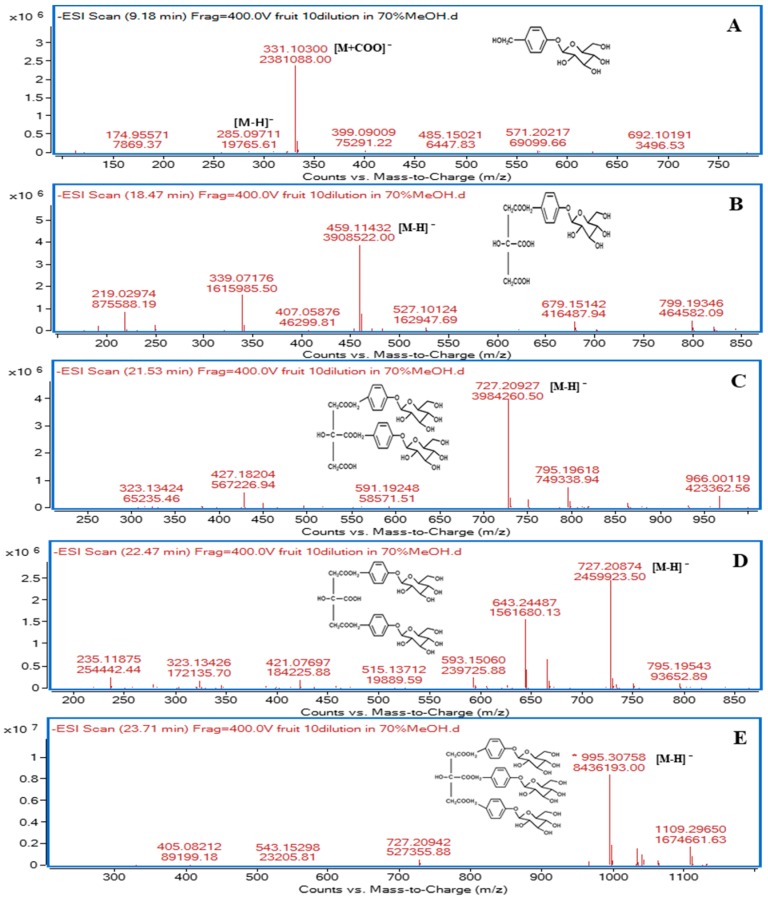
QTOF-MS spectra of parishin derivatives in *M. tricuspidata* fruit. **A**, gastrodin; **B**, parishin E; **C**, parishin B; **D**, parishin C; **E**, parishin A.

Peak **1** at a retention time of 9.20 min yielded [M + HCOO]^−^ at *m*/*z* 331.1030 and [M − H]^−^ at *m*/*z* 285.0971 (Figure 2), and the HPLC retention time was consistent with that of the authentic standard gastrodin. Therefore, this component was identified as gastrodin, a compound that has been previously reported as the main bioactive constituent of the traditional Chinese medicine, *G. elata* rhizome [27,28,29]. Peak **2** (at 13.51 min) showed a molecular ion peak [M − H]^−^ at *m*/*z* 123.0448 (C_13_H_16_O_8_), indicating that the peak corresponds to 4-hydroxybenzyl alcohol (4-HBA) and HPLC retention time was also consistent with that of the authentic 4-HBA standard. This component has also been reported previously as an important pharmacologically active constituent of *G. elata* rhizome [30,31,32] and trunk part of *M. tricuspidata* tree [11]. Peak **13** (at 23.71 min) was identified as parishin A by its characteristic fragment ion at *m*/*z* 727.2094 [M − H − gastrodin], which is derived from the deprotonated molecular ion (*m*/*z* 995.3076) and by comparison of the QTOF-MS data as well as HPLC retention time with its authentic standard. Peak **9** (at 21.54 min) and **11** (at 22.46 min) yielded molecular ion peaks [M − H]^−^ at *m*/*z* 727.2093 and *m*/*z* 727.2087, respectively. These molecular ion peaks can be considered as metabolites formed by the elimination of one gastrodin moiety (*m*/*z* 268.0971) from parishin A (Figure 2).

**Figure 3:**
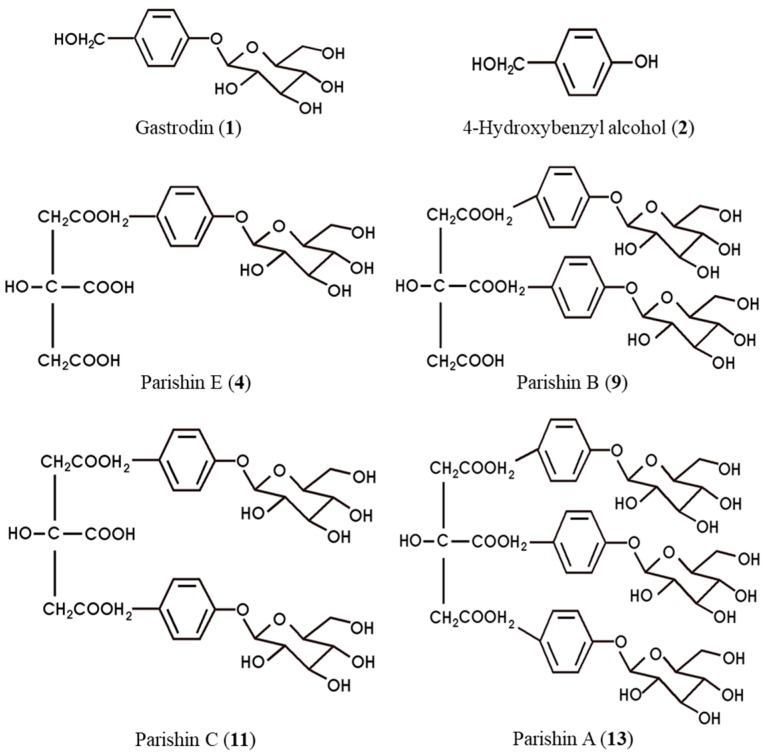
The proposed structures of parishin derivatives identified in *M. tricuspidata* fruit.

Peaks **9** and **11** were identified as parishin B and C, respectively, after comparison of the QTOF-MS data, HPLC retention times, and UV profiles with their authentic standards. Peak **4** exhibited a deprotonated ion at *m*/*z* 459.1143 [M − H]^−^ due to consecutive elimination of two gastrodin moieties (*m*/*z* 536.1942) from parishin A. This compound was confirmed as parishin E by comparison of HPLC-QTOF-MS data, retention time, and UV profile with authentic standard in HPLC analysis. The structures of these six parishin derivatives identified from *M. tricuspidata* fruit extract in this study are shown in Figure 3.

On the other hand, peak **8** showed a deprotonated molecular ion peak [M − H]^−^ at *m*/*z* 429.1398 (C_19_H_26_O_11_) and peak **15** showed a deprotonated molecular ion peak [M − H]^−^ at *m*/*z* 697.2352 (C_32_H_42_O_17_) with *m*/*z* 743.2398 [M − HCOO]^−^ and *m*/*z* 429.1397 due to loss of one gastrodin moiety from *m*/*z* 697.2352 [M − H]^−^. UV profiles (λ_max_ 220.7, 268.0 nm) of peak **8** and **15** were very similar with those of the gastrodin, parishin A, B, C and E (Table 1). These results indicate that compounds of peak **8** and **15** are also parishin analogues. Parishin A, B, C and E, which are esters formed by the condensation of citric acid and 1–3 gastrodin subunits, and its metabolite gastrodin have never been reported previously in *M. tricuspidata.*

The parishin analogues, gastrodin and 4-HBA have been recognized as important bioactive constituents in traditional Chinese medicine *G. elata* rhizome and have anticonvulsant, analgesia, calmness, hypnosis, nootropic, and anti-brain aging functions for the central nervous system [30,31,33]. The pharmacological properties of *G. elata* rhizome are mainly attributable to the presence of parishin derivatives and their metabolites [30,31,32,33]. Therefore, the parishin derivatives identified in *M. tricuspidata* fruit make the tree a potential candidate for similar therapeutic properties and applications.

### 2.3. Effects of Solvent on Extraction Yield of Parishin Derivatives

The extraction efficiency of phytochemicals from plants and plant-based foods is influenced by factors such as the chemical nature of targeted phytochemicals, polarity of solvent, extraction temperature, and extraction time. To establish effective extraction conditions for the parishin derivatives in *M. tricuspidata* fruit described above, different parameters such as solvent concentrations (aqueous methanol) and extraction methods (reflux, ultrasonication and shaking at room temperature) were investigated. The effects of different methanol concentrations on the extraction of parishin derivatives are shown in Table 2. A gradual decrease of parishin A with increase in water content, especially a sharp drop in 0% methanol, can be observed in Table 2. On the other hand, all the other compounds showed highest yields in 0% methanol. Based on the fact that parishin A is hydrolyzed to gastrodin or 4-HBA via parishins B, C and E by esterase and β-glucosidase enzymes that exist in *M. tricuspidata* fruit as the water content in the solvent system is increased [33], we concluded that the highest yield of the other compounds in 0% methanol may not be due to an actual existence of these compounds in the fruit but due to hydrolysis of parishin A during extraction. Therefore, it can be suggested that the solvent system at which Parishin A displayed most efficient extraction (60–80%) can be considered as a preferable method. Additionally, parishin A can easily break down into gastrodin or 4-HBA via parishin B, parishin C or parishin E under aqueous high−temperature condition [34]. This particular solvent between 60% and 80% methanol also resulted in a relatively higher extraction yield compared to 100% methanol, which can be related to the hydrophilic properties of parishin derivatives. 

### 2.4. Method Validation

**Figure 4:**
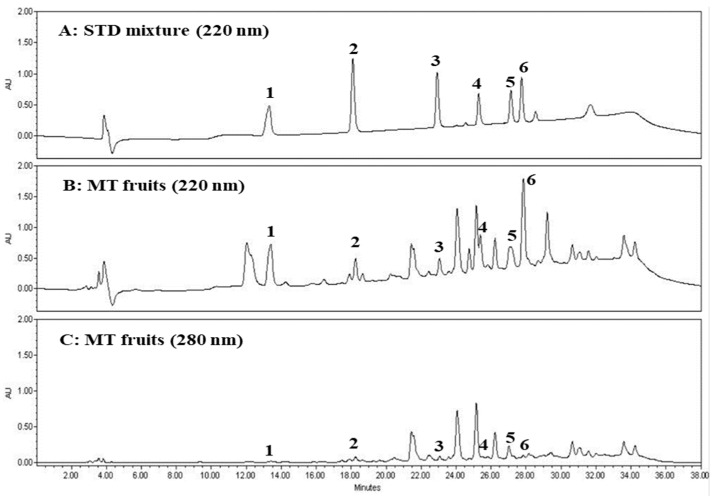
HPLC chromatograms of standard (STD) mixture of parishin derivatives (**A**) and 70% methanol extract of fully mature *M. tricuspidata* (MT) fruit at 220 nm (**B**) and 280 nm (**C**). 1, gastrodin; 2, 4-HBA; 3, parishin E; 4, parishin B; 5, parishin C; 6, parishin A.

Although spectrophotometric detection for polyphenol analysis using HPLC-DAD is generally performed in the wavelength range of 240–400 nm, parishin derivatives showed that absorption maxima (λ_max_ = 220 nm) and UV absorption was negligible at wavelengths ≥ 280 nm (Figure 4). As a result, the parishin derivatives were hardly detected at wavelengths above 280 nm in DAD. In this study, polyphenols in *M. tricuspidata* fruit were quantified by an external standard method using HPLC-DAD (220 nm). The validation data including calibration curves, *R*–square values, calibration ranges, LODs, and LOQs are shown in Table 3.

The calibration curves were shown to be linear in the range of 1.60–640 μg/mL for taxifolin, chlorogenic acid, eriodictyol, quercetin and kaempferol, 6.25–500 μg/mL for rutin and 6.25–400 μg/mL for the six parishin derivatives. The correlation coefficients for all tested standards showed good linearity (R^2^ > 0.9990) in the tested concentration ranges. The LODs and LOQs for polyphenols determined based on signal-to-noise (S/N) ratios of 3.3 and 10 were 1.343–3.287 μg/mL and 4.079–9.924 μg/mL, respectively. The LODs and LOQs for all the components were in the ranges of 0.088–0.367 μg/mL and 0.075–1.115 μg/mL, respectively. The recovery tests were performed at three different levels of 100–300 ug/g for gastrodin, parishin B, C and A. and at 75–225 μg/g for 4-HBA and parishin E (Table 3). The recoveries of gastrodin, 4-HBA, parishin C and parishin A were found to range from 100.02% to 102.94% with the standard deviations (RSDs) < 0.97%, while those for parishin E and parishin B were 98.76–99.65% with RSDs < 1.58% (Table 4). The overall RSDs of the inter-day and intra-day variations for the six components were not more than 10.70% and 9.57%, respectively (Table 5).

### 2.5. Effect of Ripening Degree on Bioactive Compounds

#### 2.5.1. External Color and Total Carotenoid

As the change in external color of *M. tricuspidata* fruit skin is easily noticeable during ripening, it is considered as one of the most accurate factors used to judge the level of maturity. It is also important to state that maturation is closely related to the quality, chemical composition, consumer acceptability, and bioavailability of the fruit [26]. The harvested fruits were classified into four different groups i.e., immature, premature, fully mature and overmature according to their color by colorimeter (Table 6) and visual judgment of external color. The a* (redness) and b* (yellowness) values increased with the progressing of maturation, while L* (whiteness) value decreased steadily. This implies that the fruit skin changed its color from green to red with maturation.

Total carotenoid content also noticeably increased in the mature stages compared to the earlier maturity stages. Especially, the total carotenoid content of the fruit was considerably higher at the fully mature (33.75 ± 1.53 mg/100 g dw) and overmature (38.09 ± 2.34 mg/100 g) than at immature (7.46 ± 0.44 mg/100 g dw) and premature (9.34 ± 0.43 mg/100 g) stages (Table 6). The remarkable increase in total carotenoid with fruit maturation is in agreement with several previous studies. It is well established that there is a linear correlation between ripening and activity of enzymes responsible for biosynthesis of carotenoids. Such correlations have been observed in fruits such as tomato [35] and acerola [36], where relative accumulation of carotenoids was observed during later stages of maturation. The significantly higher a* value (redness) in overmature compared to the lower maturity stages is mainly attributed to carotenoid pigments accumulated during ripening.

#### 2.5.2. Individual Polyphenolic Compounds

The contents of individual polyphenols at different maturity stages are presented in Table 7. The most abundant polyphenolic compounds at all four stages were chlorogenic acid (1402.43 ± 31.19–2583.11 ± 28.45 μg/g dw), eriodictyol glucoside (900.25 ± 35.43–1981.97 ± 55.78 μg/g dw) and rutin (255.16 ± 24.29–817.94 ± 22.18 μg/g dw) while most of the polyphenolic compounds, including these three compounds, significantly decreased as maturation progressed. Gradual decrease in phenolic compound contents depending on the stage of maturation might be associated with accumulation of soluble solid contents such as free sugars [37] and the reduction of primary metabolism in fruit at the fully mature and overmature stages, resulting in lack of substrates necessary for the biosynthesis of phenolic compounds [38].

Parishin A was the most abundant component of the six parishin derivatives identified in all four fruit types, followed by gastrodin, parishin E, parishin B or parishin C, and 4-HBA. It can be noticed from Table 7 that the level of parishin A changed from 9179.70 ± 93.38 μg/g dw at the immature to 6273.96 ± 27.32 μg/g dw at the fully mature stage. Similarly, the other parishin analogs showed a gradual decrease with the progressing of maturation, except 4-HBA, which showed only a slight decrease. Parishin A is an ester compound formed by the condensation of three gastrodin moieties with citric acid, which has three carboxyl groups and parishin B and C or parishin E can have one or two less moieties of gastrodin than parishin A. Therefore, parishin A can be converted into gastrodin or 4-HBA via parishin B, C or E by enzymes such as esterase during the process of fruit ripening. This might have caused a relatively sharp decrease of parishin A compared to the other compounds during the fruit maturation.

#### 2.5.3. Total Phenol and Total Flavonoid

The total phenol, total flavonoid contents, and antioxidant activities of *M. tricuspidata* fruit extracts were determined at each maturation stage. As shown in Table 7, the total phenol content of the fruit was higher at immature (140.73 ± 7.76 mg GAE/100 g dw) and premature (140.84 ± 4.58 mg GAE/100 g dw) stages, compared to that of fully mature (104.41 mg ± 4.64 mg GAE/100 g dw) and overmature (123.61 ± 2.91 mg GAE/100 g dw) stage. Total flavonoids were similarly higher at immature (155.19 ±7.78 mg QUE/100 g dw) and premature (151.94 ± 7.60 mg QUE/100 g dw) stages than at fully mature (95.71 ± 3.13 mg QUE/100 g dw) and overmature (101.83 ± 0.32mg QUE/100 g dw) stages. 

Our results are in agreement with a previous study on ripeness versus total phenol and total flavonoid contents in *M. tricuspidata* fruit by Jeong et al. [35] that reported higher total flavonoid content at unripe than ripe or over-ripe fruits with no difference observed on total phenol content among the three maturity types. Shin et al. [26] also reported a higher content of total phenol and total flavonoid at fully mature than any other maturity stages of the fruit. In the present study, both total phenol and total flavonoid content were significantly higher at immature and premature than fully mature and overmature stages. Similar findings have been reported on other fruits such as pawpaw fruit, mangoes, and Brazilian cherry [39,40,41].

#### 2.5.4. Antioxidant Activities

As the antioxidant activity of plant extracts and plant-based foods is largely affected by various factors, including the composition of the extract and assay methods, it cannot be fully evaluated by only one method. Therefore, it is usually recommended to perform more than one type of assay to obtain more reliable results. The antioxidant activities of *M. tricuspidata* fruit at four different maturity stages were determined using DPPH and ABTS free radical scavenging activities, and reducing power by FRAP assay. In order to make relative comparison of the measured values, Trolox was used as a common standard for the calibration of the methods. The results are expressed as mM Trolox equivalent (TE)/g dw. Higher TE values indicate higher antioxidant capacity in all three different assay methods. The TE values by DPPH, ABTS and FRAP assays are presented in Table 8. The DPPH assay is based on the theory that a hydrogen donor is an antioxidant and the antioxidant effect is proportional to the disappearance of DPPH free radical in the test sample. The DPPH radical scavenging activity was significantly higher (*p* ˂ 0.05) at immature and premature than fully mature and overmature stages. This is in agreement with a previous finding by Choi et al. [42], who reported a lower DPPH radical scavenging activity (as RC_50_ value) in unripe and middle ripe than ripe and overripe fruits. On the contrary, Shin et al. [26] reported higher antioxidant activity at the fully mature than at any other maturity stages.

The ABTS assay measures the relative ability of antioxidants to scavenge ABTS radical cation by reacting with a strong oxidizing agent with the ABTS salt. The reduction of the blue-green ABTS radical by hydrogen-donating antioxidants is then measured using UV-Vis spectrophotomer. The antioxidant activity based on ABTS assay was highest at immature fruit (3.91 ± 0.16 mM TE/g dw), followed by the premature (3.54 ± 0.12 mM TE/g dw), fully mature (2.99 ± 0.07 mM TE/g dw) and overmature (2.86 ± 0.07 mM TE/g dw) fruit. The antioxidant activity by the FRAP assay is measured on the basis of the ability to reduce ferric (III) ions to ferrous (II) ions. As shown in Table 8, FRAP antioxidant activity (TE value) was also significantly higher (*p* < 0.05) at immature (5.45 ± 0.06 mM TE/g dw) and premature (5.21 ± 0.09 mM TE/g), compared to fully mature (3.70 ± 0.05 mM TE/g) and overmature (4.05 ± 0.15 mM TE/g) stages. These trends are in contrast with the results obtained by Shin et al. [26], who reported strongest FRAP activity in the fully mature stage. The different results might have been caused by differences in agronomic characteristics of the plant, sorting of fruit or extraction method. On the other hand, it can also be suggested that the stronger FRAP activity in the fully mature stage is attributed to other compounds in the fruit apart from polyphenols. Similarly, we noticed an indication of direct or indirect association between contents of polyphenolic compounds and trend in antioxidant activity of the *M. tricuspidata* fruit at different maturity stages. Moreover, parishin derivatives may also have contributed to the overall antioxidant properties of the fruit.

## 3. Materials and Methods

### 3.1. Reagents

Chlorogenic acid, taxifolin, eriodictyol, quercetin, rutin, kaempferol, 4-hydroxybenzyl alcohol (4-HBA), β-carotene, 2,2-diphenyl-1-picrylhydrazyl (DPPH), 6-Hydroxy-2,5,7,8-tetramethylchromane- 2-carboxylic acid (Trolox), 2,2′-azino-bis(3-ethylbenzothiazoline-6-sulfonic acid) diammonium salt (ABTS), 2,4,6-tri(2-pyridyl)-*s*-triazine (TPTZ) were purchased from Sigma-Aldrich (St. Louis, MO, USA). Gastrodin, parishin A, parishin B, parishin C and parishin E were purchased from Chengdu Biopurify Phytochemicals Ltd. (Chengdu, Sichuan, China). HPLC grade deionized water, acetone and methanol were purchased from J.T. Baker (Center Valley, PA, USA). The other reagents used were of analytical grade and were purchased from Daihan Scientific Co., Ltd. (Wonju, Korea).

### 3.2. Plant Materials

*Maclura tricuspidata* fruit was collected in late October 2015 from plants cultivated in a farm located in Milyang district, Gyeongsangnam-do, Republic of Korea. Voucher specimen was deposited at the Herbarium of Department of Food Science and Technology, College of Agricultural Life Science, Chonbuk National University. Next, the fruits were divided into four different maturity stage groups i.e., immature, premature, fully mature and overmature based on their colors by colorimeter and visual appearance. The color was recorded using a Minolta CM3500d (Minolta Camera Co., Ltd., Osaka, Japan) tristimulus colorimeter. The results were expressed as L*, a* and b* values. The L* value indicates darkness or lightness of color and ranges from black (0) to white (100). The a* and b* values indicate color directions; +a is the red direction (+100), –a is the green direction (−80), +b is the yellow direction (+70) and –b is the blue direction (−50). The sorted fruits were freeze-dried. The samples were powdered and stored in a freezer (−20°C) until use. 

### 3.3. Sample Extraction

The powdered sample (1.0 g) was extracted with 20 mL of 70% aqueous methanol with an ultrasonicator (Hwa Shin Instrument Co., Seoul, Korea) at room temperature for 30 min and centrifuged at 4500 rpm for 15 min. The residue was extracted with 20 mL of 70% aqueous methanol followed by centrifugation as above. The supernatants were combined and evaporated under reduced pressure. The residue was typically dissolved in 10 mL of 70% aqueous methanol and diluted when necessary. In order to examine the effect of extraction solvent system on yields of parishin derivatives, powdered sample (1.0 g) was extracted in the same way as above, except different aqueous methanol concentrations (100, 80, 60, 50, 40, 30, 20 and 0%) were used.

### 3.4. Chromatographic Analysis

#### 3.4.1. HPLC-QTOF-MS

HPLC-QTOF-MS analysis was performed on an Agilent 1200 HPLC system, including a binary pump, an autosampler, and photodiode array detector (DAD) coupled to an Agilent 6550 QTOF-MS (Agilent Technologies, Palo Alto, CA, USA). HPLC analysis was performed using a ZORBAX Eclipse XDB-C18 (250 mm × 4.6 mm, 5 μm, Agilent Technologies, Palo Alto, CA, USA) at 30 °C. The mobile phase consisted of 0.1% formic acid in distilled water (solvent A), and 0.1% formic acid in methanol (solvent B). The gradient elution program was as follows: a linear gradient elution of 5–15% B from 0 to 10 min, 15–55% B from 10 to 25 min, 55–5% B from 25 to 35 min and maintained 5% B for 5 min at a flow rate of 0.8 mL/min.

The serial parameters for QTOF-MS analysis were set as follows: the nebulizer and auxiliary gas (nitrogen), drying gas (nitrogen) flow rate of 15 L/min; drying gas temperature of 225 °C; nebulizer pressure of 45 psi; sheath gas flow of 11 L/min, sheath gas temperature of 350 °C, capillary voltage of 3500 V, fragmentation voltage of 400 V, and collision energy of 0 V. The full-scan data acquisition was performed from 50 to 1200 *m*/*z* with a scan time of 1 s. All mass spectrometry data were recorded in negative ion (ESI) mode. Data was processed on the Agilent MassHunter Workstation data acquisition software (ver. B.02.01) and qualitative analysis software (ver. B. 03.01).

#### 3.4.2. HPLC

HPLC analysis was performed using an HPLC system (Waters, Milford, MA, USA) equipped with a 2690 separation module and Waters 996 DAD with an ZORBAX Eclipse XDB-C18 column (250 mm × 4.6 mm, 5 μm; Agilent Technologies, Inc., Santa Clara, CA, USA). The mobile phase consisted of 0.1% formic acid in ionized water (solvent A) and 0.1% formic acid in methanol (solvent B). The ratio of the mobile phase was maintained at A:B 95:5 (0–5 min), 85:15 (5–10 min), 45:55 (10–25 min), and 95:10 (25–40 min) at a flow rate of 0.8 mL/min. UV–Vis absorption spectra were recorded from 200–400 nm during the HPLC analysis and the quantification of individual compounds were based on peak areas at 220 nm.

#### 3.4.3. Quantification and Method Validation

Among 18 compounds identified in this study, 12 compounds were quantified by an external calibration method using chromatograms measured at 220 nm. Standards with the same structural chromophores were used to quantify the individual polyphenols. Accordingly, taxifolin and eriodictyol were used to quantify taxifolin glucoside (**1**) and eriodictyol glucoside (**6**), respectively. Quercetin derivatives (**7, 12, 16**) were quantified using quercetin and dihydrokamempferol (**17**) and kampferol glucoside (**18**) were quantified using kaempferol as a reference.

The method linearity for the quantification of each polyphenol was tested on the basis of a calibration curve, which was processed using a linear regression. The seven-point standard solutions (1.25–640 μg/mL) for polyphenols and 6.25–400 μg/mL for parishin derivatives were prepared from serial dilutions of the stock solution (1000 μg/mL) and were used for calibration according to an external method. The areas of the partially overlapped peaks were automatically calculated by the HPLC integrator. The limit of detection (LOD) and limit of quantitation (LOQ) were calculated by injecting standard solution until the signal-to-noise (S/N) ratio of each compound reached ratios of 3.3:1 and 10:1, respectively [43]. Intra-day and inter-day assays were used to determine the precision of established chromatographic conditions. The sample was extracted and analyzed in five replicates within 1 day for intra-day precision test. For the inter-day precision test, the sample was extracted and analyzed in duplicates for five consecutive days. Precisions were expressed as the relative standard deviation (RSD).

### 3.5. Determination of Total Carotenoid

Total carotenoid content was analyzed spectrophotometrically according to a method by Biswas et al. [44], with some modifications. The powdered sample (1.0 g) was extracted with 10 mL of chilled acetone under sonication for 20 min at room temperature (RT) and centrifuged at 4500 rpm for 15 min at 4 °C. The residue was re-extracted with 10 mL of chilled acetone followed by centrifugation as above. Both of the supernatants were combined and filled up to a total volume of 25 mL with acetone. Absorbance was measured at 470 nm in a UV-Vis spectrophotometer. A calibration curve was plotted using authentic standard β-carotene, and the content of total carotenoid was expressed as mg β-carotene equivalent per 100 g of dried weight (mg β-CAE/100 g dw).

### 3.6. Total Phenol and Total Flavonoid Content

#### 3.6.1. Total Phenol

Total phenol content of the sample was measured according to a method described by Chandra et al. [45], with some modifications. Briefly, 70% MeOH extract (20 μL) of each sample was mixed with 50% Folin-Ciocalteu phenol reagent (20 μL) in 96-well plates. After 5 min, 1 N sodium carbonate (20 μL) was added to the mixture and distilled water was added to adjust the final volume to 200 μL. After incubation at room temperature in the dark for 30 min, the absorbance of test sample against a blank was measured at wavelength of 725 nm using a VersaMax ELISA microplate reader (Molecular Devices, LLC, CA, USA). Total phenol content was calculated based on a calibration curve of gallic acid. The result was expressed as mg gallic acid equivalent (mg GAE)/100 g dw.

#### 3.6.2. Total Flavonoid

Total flavonoid content was measured according to a method described by Zhishen et al. [46], with some modifications. Briefly, 70% MeOH extract (30 μL) of each sample was mixed with 30 μL of 5% sodium nitrite solution. After 5 min of reaction, 300 μL of 5% aluminum chloride was added. Then 200 μL of 1 N NaOH was added 6 min later and the total volume was adjusted to 1 mL with distilled water. The absorbance of test sample against a blank was measured at wavelength of 510 nm with a Shimadzu UV-1601 spectrophotometer (Kyoto, Japan). Flavonoid content was calculated using a calibration curve of quercetin. The result was expressed as mg quercetin equivalent (mg QUE)/100 g dw.

### 3.7. Antioxidant Activity

#### 3.7.1. 1,1-Diphenyl-2-picrylhydrazyl (DPPH) Fee Radical Scavenging Activity

DPPH radical scavenging activity of the sample was determined according to a method described by Thaipong et al. [47], with some modifications. The stock solution was freshly prepared by dissolving 24 mg DPPH in methanol (100 mL) and the working solution was prepared by diluting stock solution with methanol to obtain an absorbance of 1.1 ± 0.02 units at 517 nm using the UV–Vis spectrophotometer. The 70% methanol extract (20 μL) of *M. tricuspidata* fruit was added to 80 μL working solution and 100 μL Tris–HCl buffer (0.1 M). The mixture was shaken at room temperature in dark conditions for 20 min. Absorbance was measured at wavelength of 517 nm using a microplate reader and the calibration curve was linear between 15–150 μM Trolox. The results are expressed as mM TE/g dw.

#### 3.7.2. 2,2′-Azino-bis (3-ethylbenzothiazoline)-6 sulphonic acid (ABTS) Free Radical Scavenging Activity

ABTS free radical scavenging activity was determined by methods described by Thaipong et al. [47], with some modifications. Briefly, a mixture of ABTS (7.4 mM) solution and potassium persulfate (2.6 mM) solution in 1:1 ratio was kept at room temperature for 12 h under dark conditions to form ABTS cation. The solution was diluted by adding methanol to obtain an absorbance of 1.1 ± 0.02 at 734 nm using a UV-Vis spectrophotometer. All the required solutions were freshly prepared for each assay. 100 μL of the extract was added to 1400 μL of the diluted ABTS solution and the mixture was incubated at room temperature for 2 h in the dark. After the reaction, its absorbance was measured at wavelength of 734 nm. The calibration curve was linear between 15–105 μM Trolox. Results were expressed as mM TE/g dw.

#### 3.7.3. Ferric Reducing Antioxidant Power (FRAP)

Ferric reducing power was determined using FRAP assay [48], with some modifications. The FRAP reagent was prepared by mixing 10 volumes of 300 mM acetate buffer (pH 3.6) with 1 volume of 10 mM TPTZ solution in 40 mM HCl and 1 volume of 20 mM ferric chloride solution. The sample extract (75 μL) was added to 1425 μL of FRAP reagent. The reaction mixture was incubated at room temperature for 30 min in dark conditions. Then the absorbance of the samples was measured at 593 nm. The calibration curve was linear between 15–180 μM Trolox. Results were expressed as mM TE/g dw.

### 3.8. Statistical Analysis

All experiments were performed in triplicate, unless otherwise indicated, and the results were expressed as mean ± standard deviation (SD). The statistical analysis was conducted with SPSS (ver. 10.1) for Windows and a one-way analysis of variance (ANOVA). Duncan’s multiple range tests were carried out to test any significant differences among various fruit maturity stages. Values with *p* < 0.05 were considered as significantly different.

## 4. Conclusions

Secondary metabolites in plant foods are associated with various biological activities. In fruits, the composition and contents of such metabolites may vary according to maturity levels. Although fully mature *M. tricuspidata* fruits are usually preferably edible, total phenol, total flavonoids, individual polyphenolic compounds and antioxidant activities were observed to be higher at immature and premature stages than at fully mature and overmature stages. The present phytochemical study revealed the presence of parishin derivatives such as parishin A, B, C and parishin E, and gastrodin originating from parishin analogues in this fruit for the first time in the plant. The contents of these particular compounds were also higher at immature and premature stages than at fully mature and overmature stages with a clearly noticeable decrease of parishin A observed during the maturation. Based on this, it can be concluded that parishin A and its analogues undergo significant changes during maturation of the fruit. Although *M. tricuspidata* fruit is commonly used for usual food consumption at fully mature and overmature stages, this study showed that the fruit at immature and premature stages can be considered to be preferable as raw material in the production of functional foods. These findings may add valuable information for further exploration of the fruit in terms of its therapeutic and nutritional qualities at different maturity stages.

## Figures and Tables

**Table 1:** Polyphenolic compounds identified in fully mature *M. tricuspidata* fruit by HPLC–QTOF MS.

Peak No.	tR (min)	λ_max_ (nm)	[M − H]^−^ or [M + HCOO]^−^	Molecular Formula	Error (ppm)	Identification
Phenolic and flavonoids						
3	17.75	288, 215	465.1034	C_21_H_22_O_12_	0.6	Taxifolin 3-*O*-glucoside
5	19.35	326, 216	353.0875	C_16_H_18_O_9_	1.0	Chlorogenic acid ^a^
6	19.95	287, 255	449.1084	C_21_H_22_O_11_	0.2	Eriodictyol glucoside
7	20.48	343, 264	625.1408	C_27_H_30_O_17_	0.6	Quercetin 3-*O*-sophoroside
10	22.02	353, 255	609.1456	C_27_H_30_O_16_	0.8	Rutin^a^
12	23.26	373, 258	463.0875	C_21_H_20_O_12_	0.3	Quercetin 3-*O*-glucoside ^a^
14	24.34	512	461.1663	C_22_H_22_O_11_	1.6	Peonidin 3-*O*-glucoside
16	25.43	373, 258	463.0875	C_21_H_20_O_12_	0.5	Quercetin glucoside^a^
17	27.06	365, 255	287.0559	C_15_H_12_O_6_	0.6	Dihydrokaempferol ^a^
18	27.53	363, 255	447.0927	C_21_H_20_O_11_	0.3	Kaempferol 3-*O*-glucoside ^a^
Parishin derivatives						
1	9.20	220, 268	331.1030	C_13_H_18_O_7_	1.6	Gastrodin ^a^
2	13.51	221, 273	123.0448	C_13_H_16_O_8_	1.6	4-Hydroxybenzyl alcohol ^a^
4	18.45	220, 268	459.1143	C_19_H_24_O_13_	1.8	Parishin E ^a^
8	21.15	221, 268	429.1398	C_19_H_26_O_11_	0.7	Parishin derivative-1
9	21.54	221, 268	727.2093	C_32_H_40_O_19_	0.6	Parishin B ^a^
11	22.46	221, 268	727.2087	C_32_H_40_O_19_	0.8	Parishin C ^a^
13	23.71	220, 268	995.3076	C_45_H_56_O_25_	0.2	Parishin A ^a^
15	24.83	221, 268	743.2398	C_32_H_42_O_17_	0.7	Parishin derivative-2

^a^ Positive identification by comparison of QTOF-MS data and HPLC retention time with that of an authentic standard.

**Table 2:** Extraction yields (µg/g dw) of parishin derivatives from fully mature *M. tricuspidata* fruit as extracted with different methanol concentrations.

Compounds	Methanol in Distilled Water (%)							
	100	80	60	50	40	30	20	0
Gastrodin	1055.5	1296.5	1342.3	1295.6	1272.9	2821.0	1514.8	3483.9
4-HBA	201.0	215.8	218.5	209.7	185.8	154.6	289.3	555.3
Parishin E	628.2	670.1	809.8	795.1	810.4	844.4	799.9	976.1
Parishin B	554.1	652.2	756.8	807.4	721.4	1129.0	1079.8	556.8
Parishin C	796.3	1015.2	1061.0	1019.3	949.0	994.4	976.5	1218.5
Parishin A	6155.2	7021.4	6978.2	6674.9	6625.2	6407.6	6175.7	1990.1

4-HBA, 4-hydroxybenzyl alcohol.

**Table 3:** Validation data for the representative standards used to quantify polyphenols in fully mature *M. tricuspidata* fruit using HPLC-DAD (220 nm).

Compounds	Calibration Curve	*R* ^2^	Calibration Range (μg/mL)	LOD (μg/mL)	LOQ (μg/mL)
Taxifolin	y = 34447x + 122050	0.9997	1.60–640	2.431	7.363
Chlorogenic acid	y = 32216x − 13428	0.9996	1.60–640	2.659	8.057
Eriodictyol	y = 30725x − 19052	0.9999	1.60–640	3.287	9.924
Qurecetin	y = 50655x − 49102	0.9995	1.60–640	1.343	4.079
Rutin	y = 9338x + 21264	0.9994	1.25–500	2.385	7.234
Kaempferol	y = 48264x − 50421	0.9994	1.60–640	1.752	5.316
Gastrodin	y = 8344x + 36117	0.9997	6.25–400	0.330	1.000
4-HBA	y = 34235x + 75658	0.9992	6.25–400	0.024	0.075
Parishin E	y = 23465x + 13862	0.9997	6.25–400	0.094	0.287
Parishin B	y = 10326x + 73430	0.9997	6.25–400	0.151	0.459
Parishin C	y = 12088x + 16896	0.9997	6.25–400	0.088	0.269
Parishin A	y = 15369x + 06812	0.9990	6.25–400	0.367	1.115

*R***^2^,** coefficient of determination; LOD, limit of detection; LOQ, limit of quantification.

**Table 4:** Recovery tests of six parishin derivatives.

Compounds	Original Content (μg/g)	Spiked (μg)	Observed Content (μg/g)	Recovery (%)
Gastrodin	1191.40 ± 24.81	100	1297.52 ± 24.45	100.47 ± 0.16
		200	1398.56 ± 29.88	100.51 ± 0.55
		300	1509.49 ± 24.42	101.21 ± 0.53
4-HBA	267.20 ± 11.93	75	344.58 ± 11.36	100.69 ± 0.26
		150	424.34 ± 13.03	101.71 ± 0.69
		225	506.71 ± 5.81	102.94 ± 0.92
Parishin E	335.70 ± 42.16	75	406.52 ± 44.74	98.98 ± 0.92
		150	481.26 ± 45.1	99.08 ± 1.57
		225	553.76 ± 46.58	98.76 ± 1.42
Parishin B	598.60 ± 69.71	100	692.66 ± 68.32	99.15 ± 0.21
		200	793.75 ± 67.68	99.39 ± 0.52
		300	895.47 ± 65.11	99.65 ± 0.96
Parishin C	771.30 ± 71.10	100	873.55 ± 72.85	100.25 ± 0.15
		200	974.16 ± 77.68	100.29 ± 0.62
		300	1076.14 ± 82.6	100.45 ± 0.97
Parishin A	4589.50 ± 86.39	100	4690.69 ± 80.64	100.02 ± 0.10
		200	4793.68 ± 77.56	100.08 ± 0.18
		300	4905.58 ± 73.91	100.32 ± 0.22

4-HBA, 4-hydroxybenzyl alcohol.

**Table 5:** Inter-day and intra-day tests for six parishin derivatives.

Compounds	Inter-day (*n* = 5)		Intra-day (*n* = 5)	
	Content (μg/g)	RSD (%)	Content (μg/g)	RSD (%)
Gastrodin	1197.39	3.23	1159.97	2.04
4-HBA	263.57	10.28	253.50	9.33
Parishin E	386.77	10.18	370.34	9.57
Parishin B	613.80	10.70	645.25	9.28
Parishin C	743.99	9.05	817.01	8.39
Parishin A	4737.85	1.87	4764.77	1.80

4-HBA, 4-hydroxybenzyl alcohol; RSD, relative standard deviation. Intra-day refers to five replicate experiments conducted in the same day. Inter-day refers to experiments conducted during five different days.

**Table 6:** Color and total carotenoid content at different maturity stages of *M. tricuspidata* fruit.

Maturity Stages	Color Value			Total Carotenoid ^a^
	L*	a*	b*	
Immature	77.44 ± 1.21 ^d^	3.44 ± 0.09 ^a^	31.93 ± 0.29 ^a^	7.46 ± 0.44 ^a^
Premature	70.34 ± 1.08 ^c^	15.60 ± 1.08 ^b^	33.75 ± 0.73 ^a^	9.34 ± 0.43 ^a^
Fully mature	46.77 ± 0.15 ^a^	33.84 ± 1.05 ^c^	38.29 ± 0.70 ^b^	33.75 ± 1.53 ^b^
Overmature	49.87 ± 0.72 ^b^	39.08 ± 0.11 ^d^	39.07 ± 0.41 ^c^	38.09 ± 2.34 ^c^

^a^ Total carotenoid content was expressed as mg β-CAE/100 g dw. Values are mean ± standard deviation (*n* = 3). Different superscripts in the same column indicate significant differences (*p* < 0.05). L* value indicates darkness or lightness of color and ranges from black (0) to white (100). The a* and b* values indicate color directions; +a is the red direction (+100), –a is the green direction (−80), +b is the yellow direction (+70) and –b is the blue direction (−50).

**Table 7:** Concentration of polyphenolic compounds, total phenol, and total flavonoid at different maturity stages of *M. tricuspidata* fruit.

Compounds	Immature	Premature	Fully Mature	Overmature
Gastrodin	1547.92 ± 48.29 ^b^	1857.26 ± 84.91 ^c^	945.60 ± 31.78^a^	1476.84 ± 80.73 ^b^
4-Hydroxybenzyl alcohol	ND	144.00 ± 4.711 ^b^	108.62 ± 3.06 ^a^	112.79 ± 4.34 ^a^
Taxifolin 3-*O*-glucoside	654.83 ± 24.10 ^d^	611.61 ± 17.08 ^b^	194.20 ± 4.08 ^a^	184.26 ± 1.59 ^a^
Parishin E	767.24 ± 12.27 ^c^	302.57 ± 4.80 ^b^	15.37 ± 1.17^a^	21.20 ± 1.93 ^a^
Chlorogenic acid	2583.11 ± 28.45 ^c^	2933.82 ± 2.72 ^d^	1402.43 ± 31.19 ^a^	1824.02 ± 32.70 ^b^
Eriodictyol glucoside	1981.97 ± 55.78 ^c^	2084.35 ± 74.97 ^d^	900.25 ± 34.53 ^a^	1333.94 ± 20.92 ^b^
Quercetin 3-*O*-sophoroside	114.02 ± 5.23 ^b^	136.32 ± 5.31 ^c^	95.11 ± 10.59 ^a^	109.09 ± 5.06 ^b^
Parishin B	757.20 ± 48.46 ^c^	606.04 ± 32.47 ^b^	31.19 ± 3.65 ^a^	86.17 ± 14.59 ^a^
Rutin	817.94 ± 22.18 ^c^	798.27 ± 10.1 ^c^	417.38 ± 8.84 ^b^	255.16 ± 24.29 ^a^
Parishin C	858.38 ± 36.22 ^d^	628.26 ± 15.41 ^b^	497.00 ± 17.02 ^a^	760.49 ± 29.88 ^c^
Quercetin 3-*O*-glucoside	438.7 ± 11.9 ^c^	498.5 ± 11.9 ^d^	284.5 ± 14.1 ^b^	211.7 ± 3.7 ^a^
Parishin A	9179.70 ± 93.38 ^c^	9306.55 ± 49.60 ^c^	6273.96 ± 27.32 ^a^	7387.28 ± 99.37 ^b^
Dihydrokaempferol	52.72 ± 1.18 ^b^	47.26 ± 2.47 ^b^	30.09 ± 2.00 ^a^	75.18 ± 5.31 ^c^
Kaempferol 3-*O*-glucoside	124.68 ± 5.44 ^a^	71.29 ± 1.98 ^c^	23.77 ± 1.37 ^a^	34.80 ± 0.81 ^b^
Total phenol	140.73 ± 7.76 ^c^	140.84 ± 4.58 ^c^	104.41 ± 4.64 ^a^	123.61 ± 2.91 ^b^
Total flavonoid	155.19 ± 7.78 ^b^	151.94 ± 7.60 ^b^	95.71 ± 3.13 ^a^	101.83 ± 3.02 ^a^

ND, not detected. Values are the mean ± standard deviation (*n* = 3). Individual compounds are expressed in μg/g dw. Total phenol content is expressed in mg GAE/100 g dw. Total flavonoid content is expressed in mg QUE/100 g dw. Different superscripts in the same row indicate significant differences (*p* < 0.05).

**Table 8:** Antioxidant activities (mM TE/g dw) at different maturity stages of *M. tricuspidata* fruit.

Activities	Immature	Premature	Fully Mature	Overmature
DPPH	6.70 ± 0.06 ^d^	6.02 ± 0.15 ^c^	5.40 ± 0.10 ^b^	4.54 ± 0.23 ^d^
ABTS	3.91 ± 0.16 ^c^	3.54 ± 0.12 ^b^	2.99 ± 0.07 ^a^	2.86 ± 0.07 ^a^
FRAP	5.45 ± 0.06 ^d^	5.21 ± 0.09 ^c^	3.70 ± 0.05 ^a^	4.05 ± 0.15 ^b^

Different superscripts in the same row indicate significant differences (*p* < 0.05).
